# Optimising Control Device Luring Strategies for Invasive Predator Control: A Modelling Approach

**DOI:** 10.1002/ece3.70604

**Published:** 2024-11-28

**Authors:** Giorgia Vattiato, Patrick M. Garvey, Rachelle N. Binny, Michael J. Plank, Andrew M. Gormley, Graham J. Hickling

**Affiliations:** ^1^ Manaaki Whenua – Landcare Research Lincoln New Zealand; ^2^ University of Canterbury Christchurch New Zealand

**Keywords:** invasive species, kill‐traps, lure change, pest eradication, trap‐shyness

## Abstract

Invasive predators pose a serious threat to native biodiversity, with trapping being one of several methods developed to manage and monitor their populations. Many individuals in these predator populations have been found to display trap‐shyness, which hinders eradication and results in inaccurate estimates of population size. Lures are used to help overcome trap‐shyness by increasing the probability of interaction with the device, but the extent of trap‐shyness in wild populations, and the best timing for the introduction of a new lure or combination of lures, are uncertain. A key challenge for wildlife managers is maximising the efficacy of invasive predator control, particularly in relation to baiting and trapping, so that pests are extirpated, or survivors are reduced to a minimum. We first use a Bayesian estimation method to quantify trap‐shyness in a population of brushtail possum ( *Trichosurus vulpecula* ) in a New Zealand forest; the resulting estimated parameters are then used to calibrate a stochastic, individual‐based model simulating the outcomes of different luring scenarios. We show that the brushtail possum population analysed was likely split into a smaller, very trappable group and a larger trap‐shy group, with a low mean nightly probability of interaction with traps of 0.28 [0.14–0.56]. Furthermore, our results show that under the assumption of independent attraction levels towards different lures, using a combination of lures simultaneously can result in a greater and faster population knock‐down than using a single lure, or than to switch from one lure to another. The model presented can be used to infer wildlife population trappability from capture data, and our simulation results highlight the potential of improved luring strategies to capture individuals in post‐control residual populations.

## Graphical abstract

Invasive predators pose a threat to native biodiversity, and their management is hindered by trap‐shyness. Using Bayesian estimation, researchers quantified trap‐shyness in wild brushtail possums, finding a large number of trap‐shy individuals with a low probability of trap interaction. The study suggests that employing multiple lures throughout a trapping operation is more effective than switching lures for reducing possum populations.

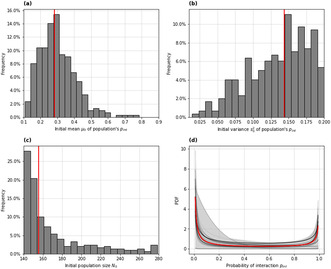

## 1 Introduction

Animal personality has been shown to influence characteristics that are important in a pest management context such as dietary preferences (Mella et al. 2015), reproduction (Réale et al. 2000), dispersal (Bremner‐Harrison, Prodohl, and Elwood 2006) and risk‐taking behaviours (Mella et al. 2015). Eradicating invasive wildlife populations is made particularly challenging by some individual animals' avoidance of monitoring or control devices, a phenomenon often referred to as ‘recalcitrance’ or ‘trap‐shyness’ (Johnstone 2021; Linhart et al. 2012; Thompson 1953). Individual characteristics of a pest population, such as those related to personalities, demographic factors or body condition, can contribute to trap‐shyness and kill‐trapping increases the proportion of trap‐shy survivors in the population (Johnstone, Garvey, and Hickling 2023; Seymour et al. 2005; Vattiato et al. 2021). Determining the prevalence of trap‐shyness within a pest population is challenging, however different possible personality distributions can be explored using scenario modelling (Vattiato 2021).

Managing pests with standard traps is economical and can effectively target most pests, yet once captures reach an asymptote, the presence of trap‐shyness suggests that additional rounds of trapping will achieve negligible population reductions. It is important to understand the distribution of trap shyness for several reasons. First, density‐impact functions (DIFs) describe the relationship between residual pest abundances and biodiversity outcomes and can reveal the degree of vulnerability of native species to changes in pest density (Norbury et al. 2015). Where managers have specific pest density targets, they need to understand the distribution of trap‐shyness, as alternative methods (e.g., conservation dogs) will be required to achieve target densities if there is a remnant population of trap‐shy individuals. Second, behavioural traits associated with trap‐shyness—such as neophobia, low activity and low exploration—have recently been identified in possums and stoats (Johnstone, Garvey, and Hickling 2023). These traits of personality have direct ecological consequences (e.g., prey specialisation) and implications for control operations targeting survivors. As some traps target different spectrums of personality than others (Johnstone, Price, and Garvey 2024), understanding the distribution of trap shyness is essential to inform adaptive management approaches. In addition, trap catch data inform management strategies and provides demographic information on the targeted pests. However, population estimates based on trap catches will be inherently biased, as captures will be skewed towards bolder, risk‐taking individuals (Vanden Broecke et al. 2021). If there is lack of information about the distribution of trap‐shyness, any decisions based on capture data could be problematic. Finally, survivors can be targeted more effectively with passive devices (e.g., leg hold trap or conservation dogs). However, these approaches require daily trap checking and are more expensive than standard approaches. Given the greater cost associated with these interventions, managers need to minimise the number of survivors which requires an understanding of the distribution of trap‐shyness in the remnant population.

The probability that an individual will interreact with a trap is a product of the animal's intrinsic fear of the device, the value of the lure to the animal and the animal's missed opportunity cost (MOC) (Garvey et al. 2020). Variations in MOC can alter an individual's susceptibility to capture (e.g., a food lure when prey resources are limited), while applying more attractive lures can overcome reluctance to engage due to fear of devices. While highly attractive lures may attract most pests in a population, variation in individual dietary preferences suggests that no single lure will target all animals. Indeed, the value of a lure may change for the same individual over time (e.g., oestrous female odour during the breeding season), so that individuals exhibit variation in trappability through time (Rhoades, Best, and Stachowicz 2018). In addition, different lures can be more or less effective in overcoming trap‐shyness: nonfood lures are preferred when food is abundant, such as in low‐density populations (Clapperton, Murphy, and Razzaq 2017; Garvey et al. 2017), whereas food baits with higher fat (for rats) or protein (for possums) compositions can outperform their less nutritious counterparts (Jackson, Hartley, and Linklater 2015; Russell, Towns, and Clout 2008). In addition, there is evidence of dietary preferences between individual animals (Esposito, Ceresa, and Buoli 2021; Herath et al. 2021) and of different individuals preferring one lure over another (Glen et al. 2023). This suggests that a combination of lures might be able to target a wider proportion of the population and be the most efficient strategy for achieving eradication in kill‐trapping control programmes. While deploying all lure types simultaneously may seem optimal, there are associated costs that can limit this approach. Lures, such as food baits or social lures, degrade at different rates so the attractiveness can vary across time. An optimal approach may deploy long life lures at the outset, to minimise rebaiting schedules (i.e., costs) and maximise the duration of attraction. Furthermore, targeted pests may become desensitised to lures if all lure types are deployed simultaneously. Another strategy could be to deploy lures in order of effectiveness, so targeted pests are only exposed to a single lure at a time, especially where responses to lures are independent of one another. Deploying all lures simultaneously may also risk that pests generalise their response to lures and avoid all devices. Generalisation occurs when animals recognise stimuli sufficiently similar or comparable to a known cue and may respond in a similar fashion (Price et al. 2020). This suggests that the qualities of the lure, and similarity to other lures, may influence the optimal deployment approach to limit the risk of generalisation.

In this paper, we use a spatially explicit, individual‐based model to simulate the dynamics of a predator population exhibiting heterogeneity in degree of trap shyness during a kill‐trap eradication program. We use a simple Bayesian inference method to calibrate key model parameters associated with heterogeneity in trap‐shyness, by fitting the model's predicted number of captures over time to a dataset of possum captures in a New Zealand forest. Under the optimistic assumption that attraction to different lures is independent (the response of a trap‐shy individual to one type of lure does not influence their response to another), we then use the calibrated model to compare the effects of introducing a secondary lure at various points during the kill‐trap operation to a single‐lure baseline scenario.

## 2 Methods

We use a stochastic, individual‐based model including density‐dependent reproduction, mortality and density‐dependent home‐range radii, similar to that described in Vattiato (2021), where this model was also used to study the effect of vertical transmission of trap‐shyness, and the link between home‐range use and encounter probability. First, we calibrate the model using data from a pest removal experiment conducted using kill‐traps to remove invasive brushtail possums ( *Trichosurus vulpecula* ) from a New Zealand conservation reserve where the overabundant possum population was impacting native species biodiversity. The calibration process is used to estimate the precontrol population size, as well as the distribution of the trap‐shyness trait within that population. Next, we use the calibrated model to simulate seven scenarios of an 8‐month‐long kill‐trap operation, each using a different combination of lures. The simulation outcomes are then used to compare the effectiveness of the different luring strategies.

All model simulations were conducted using custom scripts coded in Python 3.1 (see Data Availability Statement), based on the model specifications described later in this section.

### 2.1 Study Area and Trap Grid

The trapping experiment is described in detail in Johnstone, Garvey, and Hickling (2023). Briefly, possums were trapped from a 120 ha reserve of old‐growth beech/podocarp forest in North Canterbury, New Zealand (42^o^33′ S, 173^o^05′ E) that was fenced to exclude livestock that grazed on surrounding pastures. Prior to the experiment, there had been no significant trapping in the reserve in recent years, with possum sign found throughout the reserve at levels suggesting possum abundance was near the ecological carrying capacity of the habitat.

**FIGURE 1:**
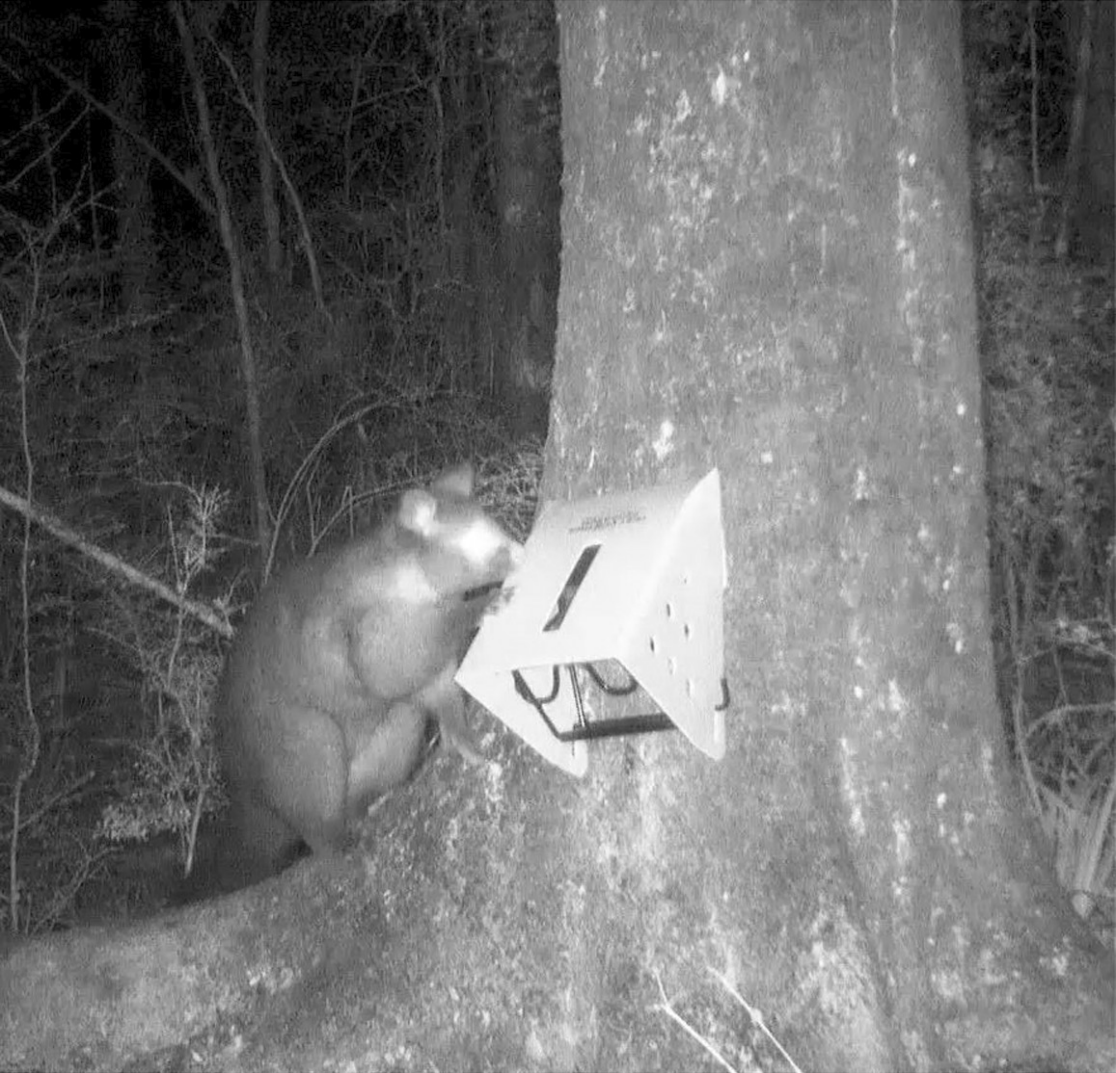
Example of a brushtail possum interacting with a Sentinel kill‐trap deployed in the forest reserve.

In early May 2021, 66 possum ‘head‐in’ kill‐traps (Sentinels(TM) and Trappinators(TM)) were installed on transects within the reserve (Figure S1). These transects followed forest margins, ridgelines and other landscape features expected to favour possum movement and trap encounters. Traps were attached to tree trunks c. 50 cm above ground level (Figure 1). Possums that inserted their head through an opening in the trap to access bait triggered a lethal kill‐bar. Traps were baited with peanut butter placed on the trigger mechanism and lured with mixture of flour and icing sugar smeared on the tree trunk below the trap. The traps were not self‐resetting, so regular visits by field staff were required to clear carcasses and re‐set sprung traps.

Additional kill‐traps were deployed in June and July 2021, resulting in the eventual deployment of 105 active traps (corresponding to ~0.9 traps per ha) across the entire reserve (Figure S1). The expected home range of possums in eastern forest habitat is c. 1–2 ha (Cowan and Glen 2021), so this trap coverage provided high confidence that all possums within the reserve would have at least one kill‐trap within their home range. Initially, traps were checked and reset twice a week. As possum numbers declined and catch rates fell, the interval between checks lengthened progressively. Johnstone, Garvey, and Hickling (2023) reported on catches up until January 2021; trapping continued for several years thereafter.

The reduction in possum abundance in the reserve achieved by kill‐trapping was assessed using several indices of relative possum abundance. Kill‐trap catch rate was monitored but it was recognised that trap‐shyness would increasingly bias this rate. Therefore, trail camera and chew‐card surveys were also implemented prior to the start of trapping and again in June and September 2021. At three time‐points during the study cage traps and padded leg‐hold traps were briefly used to collect samples of live possums from the reserve; these capture data were included in the analysis. Collectively, these several indices were consistent in suggesting that possum numbers in the reserve had been substantially reduced by October 2021 (see table 1 in Johnstone, Garvey, and Hickling 2023).

In the model described below, the study area was simplified to a 175 ha rectangle encompassing the trap layout, with a 100 m buffer from the outermost traps in each direction. Simulated possum home ranges were randomly placed within this rectangle.

### 2.2 Simulation Model

#### 2.2.1 Density‐Dependent Reproduction and Natural Mortality

Possum populations show density‐dependent effects on fecundity (Cowan and Glen 2021; Ramsey et al. 2002). Using daily time steps, we model the birth rate at time *t* as

(1) $$B_{t}=\left(\alpha -cN_{t^{*}}\right)\;f\left(t\right)$$

where α is the maximum per‐capita annual birth rate, *c* is a coefficient representing the strength of density‐dependent effects on birth rate (see below) and $f\left(t\right)$ corresponds to the normal probability density function used to model the reproduction season, evaluated at day *t* and truncated to [0, 365] days. To account for the gestation period, we update the density‐dependent term $N_{t^{*}}$, that is, the number of live adults at time $t^{*}$, the beginning of the reproduction season, estimated at the 1st of February (Table 2).

Brushtail possums in New Zealand have been observed to have one major breeding season starting at the end of summer and lasting for 3–4 months (Cowan and Glen 2021; Crawley 1973; Lustig et al. 2019). A small portion of females have a second breeding season in spring. For simplicity, we ignored the second breeding season and modelled reproduction season as a normal distribution centred around the first of April and with a 20‐day standard deviation (Table 2). Note that we include reproduction in our model for generality, but our simulations are limited to an 8‐month period starting immediately after the end of a breeding season, hence reproduction will have a negligible effect on population dynamics in the scenarios presented in this paper.

Natural mortality is modelled as a constant per capita mortality rate per unit time using the formula

(2) M=1/l

with l being average life expectancy.

The expected number of newborns $J_{t+1}$ and natural deaths $D_{t+1}$ on a given day t+1 is then calculated as

(3) $$J_{t+1}\sim \text{Binomial}\left(N_{t},B_{t}\mathrm{\delta t}\right)$$

(4) $$D_{t+1}\sim \text{Binomial}\left(N_{t},\mathrm{M\delta t}\right)$$

where δt=1 day is the time step.

Newborns do not affect the density‐dependent fecundity until they are 1 year of age (Cowan and Glen 2021), but they can die naturally or from trapping at the same rates as adults.

Note that in the absence of trapping the model behaves like a stochastic density‐dependent model with seasonal reproduction (Figure S3, top). The population size will be at statistical equilibrium when $\alpha -cN_{t^{*}}=M$. This implies that the value of the coefficient c must equal $\left(\alpha -M\right)/\left(\mathrm{AK}\right)$ where A is the size of the study area and K is the carrying capacity for population density.

The study area A for the model fitting simulations is given an arbitrary value of 175 ha (Table 2). The carrying capacity K=9 possums ha^−1^ is taken from the values allocated to New Zealand land cover classes by Warburton, Cowan, and Shepherd (2009), corresponding to a noncontrolled population of possums in a mixed beech‐podocarp‐broadleaved forest. The value of α is set by noting that α−M is the net rate of per capita population growth in the low‐density limit, a quantity for which estimates are available in the literature for brushtail possums in New Zealand (Cowan and Glen 2021).

#### 2.2.2 Probability of Capture

The probability of an animal being captured or detected by a device is partitioned into two components: the probability that the animal encounters a device within its home‐range area, and the probability that it interacts with and triggers that device given an encounter. For brushtail possum, the probability of an animal encountering a device is inversely related to its home‐range size (Ball et al. 2005). This relationship depends on several factors including the trap grid spacing, trap and bait type, the animal's denning behaviour, movement rate and perception distance (Vattiato 2021). For the model fitting procedure, we use the same trap grid layout as the one used in the field trial (Johnstone, Garvey, and Hickling 2023). For the scenario simulations, we use a generic homogeneous landscape of fixed area A=100 ha overlaid with a square grid of traps with distance $d_{\text{traps}}=100$ m between traps (Table 2).

We define the nightly capture probability by one device $p_{c,i}\left(d\right)$, that is, the probability that an individual i encounters and triggers a device set at a distance d from its home‐range centre, in a single night, as

(5) $$p_{c,i}=p_{\mathrm{enc}}\left(d\right)p_{\mathrm{int},i},$$

where $p_{\mathrm{enc}}\left(d\right)$ is the probability that the individual encounters a device at distance d from its home‐range centre, and $p_{\mathrm{int},i}$ is the probability that the individual interacts with the device, given encounter. We model population heterogeneity at the individual level, by assigning a different probability of interaction $p_{\mathrm{int},i}$ to each individual i in the population, with each $p_{\mathrm{int},i}$ value drawn from a Beta distribution (distribution bounded between 0–1 and commonly used to draw random probabilities) with mean $\mu_{0}$ and variance $\sigma_{0}^{2}$. We infer these last two parameters by fitting our simulation model to capture data, as described in the next section.

The value $p_{\mathrm{enc}}\left(d\right)$ of the probability of encountering a single trap at a distance d from the home range centre is not well described in the literature. However, we can derive it from Equation (5) and the well‐established relationship $p_{c}\left(d\right)=g_{0}e^{-d^{2}/2\sigma^{2}}$ (Efford, Borchers, and Byrom 2009). Ideally, we would have a measure of $g_{0}$ for each individual in a population, to account for individual differences in trappability. However, as estimates of $g_{0}$ reported in the literature do not distinguish between individuals, we assume $p_{\mathrm{int}}=\mu_{0}$ in Equation (5) to derive Equation (6), with $\mu_{0}$ being the mean of the distribution of initial $p_{\mathrm{int}}$ values for our simulated population, prior to control (Table 2):

(6) $$p_{\mathrm{enc}}\left(d\right)=\frac{g_{0}}{\mu_{0}}e^{-d^{2}/2\sigma^{2}}$$

where $g_{0}$ is the average nightly probability of capture at the home‐range centre (i.e., $g_{0}=p_{\mathrm{enc}}\left(0\right)\mu_{0}$).

The spatial decay parameter σ is a measure of the individual's home‐range size: assuming an animal occupies its home range, on average, according to a symmetric bivariate normal distribution, then the area the animal occupies 95% of the time is a circle of radius 2.45σ with area $\pi {\left(2.45\sigma \right)}^{2}$ (Efford 2004). Estimates of $g_{0}$ and σ can be obtained by fitting spatially explicit capture–recapture (SECR) models to capture–recapture data (Borchers and Efford 2008; Efford and Fewster 2013), and estimates for brushtail possum populations have been reported for a range of population densities and different environmental conditions (Vattiato et al. 2023). For brushtail possums, there is an inverse relationship between $p_{\mathrm{enc}}\left(0\right)$ (and therefore $g_{0}$) and σ (Anderson et al. 2021; Sweetapple and Nugent 2018; Vattiato et al. 2023), resulting from animals with larger home‐range areas spending, on average, less time at the home‐range centre than animals with a smaller home range, and which for brushtail possums in New Zealand is best described by the power law $g_{0}=5.67{\left(\sigma /\sigma_{0}\right)}^{-0.99}$ for each individual i, with $\sigma_{0}=1m$ (Vattiato et al. 2023). See Figure S2 for some graphical examples of the relationship between $p_{\mathrm{enc}}\left(d\right)$ and σ. There is also an inverse relationship between σ and population density D of possums (Anderson et al. 2021; Efford et al. 2016), best described by the function $\sigma =73.7{\left(D/D_{0}\right)}^{-0.4}$, with $D_{0}=1ha^{-1}$ (Vattiato et al. 2023).

At the beginning of each simulation, the coordinates of each animal's home‐range centre are randomly drawn from an array of possible locations. This position is used to calculate the distance $d_{\mathrm{ij}}$ to each trap j located within the home‐range area of individual i and the associated probability of the animal encountering trap j in a single night $p_{\mathrm{enc},i}\left(d_{\mathrm{ij}}\right)$ using Equation (6). The probability $p_{\text{encTOT},i}$ that individual i encounters at least one of the traps in its home range in a single night $p_{\text{encTOT},i}$ is then calculated as follows:

(7) $$p_{\text{encTOT},i}=1-\prod_{j=1}^{J}\left(1-p_{\mathrm{enc},i}\left(d_{\mathrm{ij}}\right)\right)$$

where J is the total number of traps in the home range. These calculations are done independently for each individual, and while the randomly drawn home‐range centres can result in an overlap of home ranges, we assume that each animal's $p_{\text{encTOT}}$ is not affected by the position of other animals' home ranges. Figure S3 illustrates how an increase in population size corresponds to a decrease in average home‐range radius, and consequently a decrease in the population's average total probability of encounter ${\hat{p}}_{\text{encTOT}}=\frac{1}{N_{t}}\sum_{i}p_{\text{encTOT},i}$.

### 2.3 Parameter Inference and ABC Model Fitting

We first use an approximate Bayesian computation (ABC rejection) approach (Beaumont 2010; Sunnaker et al. 2013) to fit our population model to the Lottery Bush field data described previously. This is done to estimate three critical model parameters: the distribution of the trap‐shyness trait, described by the mean $\mu_{0}$ and variance $s_{0}^{2}$ of the Beta distribution from which the individual $p_{\mathrm{int}}$ values were drawn at the start of the simulation, and the initial population size $N_{0}$, with prior distributions defined in Table 2. This approach finds combinations of parameter values for which the model output is approximately consistent with the time series data on captures and rejects combinations of parameter values that are inconsistent with the data. Intuitively, the level of heterogeneity in the population is inferred from the pattern of captures over time. Low levels of heterogeneity are associated with an exponential decay in the number of captures, and high levels of heterogeneity are associated with subexponential decay (i.e., a decay rate that decreases over time).

The prior distributions chosen for the mean of $p_{\mathrm{int}}$ were uninformative (see Table 2), as there is little to no information on the magnitude of interaction probabilities in wild possum populations. The prior for the variance of $p_{\mathrm{int}}$ was chosen to be mildly informative with an upper bound at 0.2 to limit the range of extreme scenarios. The prior chosen for the initial population size $N_{0}$ was left‐bounded with the total number of captures from the Lottery Bush data, and right‐bounded with double that number of captures. This assumes that at least 50% of the Lottery Bush population was caught by the end of the trapping operation, which is a reasonable assumption considering the large reduction seen in multiple indices of possums' relative abundance (Johnstone, Garvey, and Hickling 2023).

For each combination of parameter values, randomly drawn from their prior distribution, we run the model and calculate an error function, defined as

(8) $$f\left(x,y\right)=\frac{1}{n}\sum_{t_{\text{DATA}}}{\left(x_{t}-y_{t}\right)}^{2}$$

With $t_{\text{DATA}}$ being the timestamps of the available capture data, $x_{t}$ being the modelled captures at time t and $y_{t}$ being the observed captures at time t. We ran the model for 30,000 parameter combinations and retained the 300 combinations corresponding to the 1% smallest errors $f\left(x,y\right)$ (similarly to the ABC procedures described in Lustig et al. 2023; Browning et al. 2018) to produce an approximate unimodal posterior distribution.

### 2.4 Change of Lure Scenarios

The calibrated population model is then used to explore the effect of deploying different combinations of lures, and different timings for the change to a new lure on the success of a trapping operation. These simulations assume independence in the heterogeneity of responses to different lures, as explained further in this section. We simulate seven different scenarios, with two different catch rate thresholds (i.e., the ratio of the current catch rate over the initial catch rate that triggered a change in lure), as defined below, and two kinds of ‘new lure’ (Table 1).

**TABLE 1:** Description of the seven simulation scenarios run to test the effect of using a combination of lures over using a single lure in a kill‐trap operation. Note that the secondary lure attractiveness is given as a multiplier of the initial population's (precontrol) mean probability $\mu_{0}$ of interaction with a lure A device. For these simulations, $\mu_{0}$ is randomly drawn from the posterior distribution of parameters, as described in the previous subsection.

Scenario	Lures	Secondary lure attractiveness (mean of initial population's $p_{\mathrm{int}}$ beta distribution)	Change of lure threshold $r_{c}$ (% drop in daily captures)
Baseline (single lure)	A	—	—
Combination lure 1	A + B	$\mu_{0,B}=\mu_{0}$	—
Lure switch 1.1	A, then B	$\mu_{0,B}=\mu_{0}$	80%
Lure switch 1.2	A, then B	$\mu_{0,B}=\mu_{0}$	90%
Combination lure 2	A + C	$\mu_{0,C}=2\mu_{0}$	—
Lure switch 2.1	A, then C	$\mu_{0,C}=2\mu_{0}$	80%
Lure switch 2.2	A, then C	$\mu_{0,s}=2\mu_{0}$	90%

We run 10,000 simulations for each scenario using parameter values drawn from the posterior. For each scenario, we then report the median and 95% credible intervals for the final population size and the mean $p_{\mathrm{int}}$ of the final population.

The baseline scenario corresponds to one where we keep applying the default lure (lure A) for the entire duration of the trapping programme. Individuals' $p_{\mathrm{int}}$ values for interaction with lure A are randomly drawn from the best fitting distribution obtained through the ABC model fitting, and they remain constant until the end of the simulation.

For the lure combination scenarios, we draw two independent values of $p_{\mathrm{int}}$ for each individual, one for lure A, and one for the ‘new lure’. The independence of the two $p_{\mathrm{int}}$ values is an arbitrary modelling choice corresponding to a best‐case scenario. At each time step, individuals have some probability of encountering a trap with either of the two lures, dependent on the lure combination layout (described in the next paragraph) and a lure‐dependent probability of capture given by $p_{c,i,L}=p_{\text{encTOT},i}p_{\mathrm{int},i,L}$, with $p_{\mathrm{int},i,L}$ being the probability of interaction assigned to individual i for lure L. Note that the $p_{\mathrm{int},i,L}$ values for each lure are drawn independently from each other, which means that an individual could be very trap‐shy towards lure A, but very attracted towards the second lure, and vice versa. In other words. B is simulated to have the same average attractiveness as lure A, but will attract a different subset of the population. This approach accounts for individual preferences and variation in risk‐taking behaviour.

We also tested two different ‘new lures’ (called B and C), one corresponding to a distribution of $p_{\mathrm{int}}$ identical to that for lure A (beta distribution with mean $\mu_{0}$, standard deviation $\sigma_{0}$), and a more attractive one corresponding to a distribution of $p_{\mathrm{int}}$ with mean $2\mu_{0}$ and standard deviation $\sigma_{0}$, that is, double the mean and equal variance of that for lure A.

We considered two different luring scenarios: one where the two lures (default and new) are present in equal numbers for the entire duration of the programme (each individual has a 50% probability of encountering either on any night), and one where all lures are switched from the default to the new lure once the ratio of the current daily catch rate (averaged over the past 7 days to correspond with standard trapping practice) over the initial daily catch rate drops by a set amount $r_{c}$ (Table 1). Note that for the scenario where both lures are present concurrently, we do not explicitly model the position of each lure. Instead, for simplicity, we assume that when an individual encounters a trap, it contains the default lure with probability 0.5 and the new lure with probability 0.5, independent of previously encountered lures.

## 3 Results

### 3.1 Model Fitting

**FIGURE 2:**
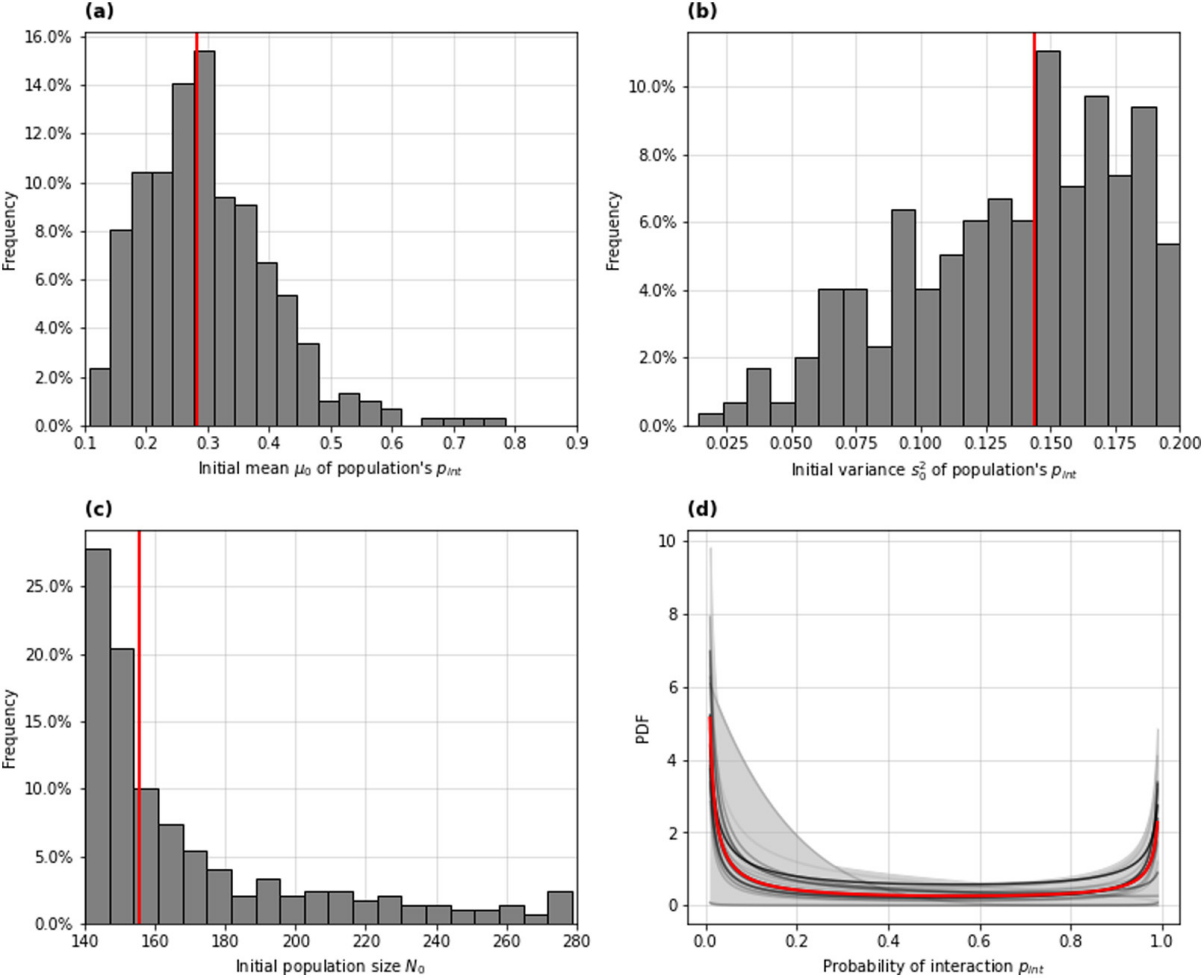
Histograms showing the marginal posterior distributions of each of the three fitted parameters (a, b, c) for the 300 accepted model runs (1% best runs resulting from 30,000 random draws from the prior [Table 2]), with the best fitting parameter set indicated in red. (d) Posterior Beta distributions, including the distribution function corresponding to the median $\mu_{0}$ and $s_{0}^{2}$ (red line), used to randomly draw the individuals' probabilities of interaction $p_{\mathrm{int}}$ at the beginning of each scenario simulation, a credible interval enveloping the distributions corresponding to the 1% best fitting parameter sets (grey shaded area), and a sample of 20 posterior beta distribution functions (grey lines, with a transparency gradient which reflects the goodness of fit of the corresponding posterior parameter set—the darker the line, the smaller the associated error).

The simulation model resulted in a reasonable fit to the Lottery Bush capture data (Figure S4). The marginal posterior distributions for the mean $\mu_{0}$ and variance $s_{0}^{2}$ parameters of the beta distribution for the initial population's $p_{\mathrm{int}}$ had median and 95% credible interval values $\mu_{0}=0.28$ [0.14, 0.56] and $s_{0}^{2}=0.14$ [0.04, 0.20], corresponding to populations made up of mostly trap‐shy. Both these sets of parameter values correspond to a ‘U‐shaped’ Beta distribution for the probability of interaction $p_{\mathrm{int}}$ (Figure 2d), meaning that most individuals in the initial population had interaction probabilities that were either very low (‘trap‐shy’ individuals) or very high (‘trappable’ individuals). A comparison of model results and fitted data can be found in Figure S4.

The marginal posterior distribution for the initial population size had a median and 95% credible interval values of $N_{0}=156$ [141, 268], the distribution was strongly left‐skewed (Figure 2c), indicating that most of the population had been caught during the trapping operation at Lottery Bush.

**TABLE 2:** Parameter values used in the population dynamics simulations for the exploration of the effects of trap‐shyness on pest eradication. The ‘Values’ for the initial population's $p_{\mathrm{int}}$ beta distribution and initial population size correspond to the priori uniform distribution used in our model fitting procedure.

Parameters	Symbol	Value	Comments/references
*Landscape*			
Study area	*A*	175 ha	Model fitting simulations use A = 1160 m × 1660 m to encompass trap layout from (Johnstone, Garvey, and Hickling 2023)
Square trap grid spacing	$d_{\text{traps}}$	100 m	Model fitting simulations use the same trap layout as in (Johnstone, Garvey, and Hickling 2023)
Carrying capacity	*K*	9 possums ha^−1^	(Warburton, Cowan, and Shepherd 2009), value corresponding to populations of possum in New Zealand's mixed beech‐podocarp‐broadleaved forests
*Population*			
Trap perception distance		10 m	Assumed
Lifespan in the absence of control measures	l	13 years	Cowan (2001)
Annual birth rate	α	0.77	Calculated as annual growth rate in Cowan and Glen (2021) + annual mortality rate from Equation (2)
Peak of reproduction season (mean of f(t))		1st April	Assumed
St. dev. of reproduction season function f(t)		20 days	Assumed
Maximum home‐range radius		380 m	Corresponding to the maximum value of σ (155 m) found in Vattiato et al. (2023)
Nightly probability of capture at home‐range centre	$g_{0}$	Variable	See text in ‘Probability of capture’
Home‐range spatial decay parameter	σ	Variable	See text in ‘Probability of capture’
Initial mean of default population's $p_{\mathrm{int}}$ beta distribution	$\mu_{0}$	$\sim U\left[0.1,0.9\right]$	Fitted
Initial variance of default population's $p_{\mathrm{int}}$ beta distribution	$s_{0}^{2}$	$\sim U\left[0.01,0.2\right]$	Fitted
Initial population size	$N_{0}$	$\sim U\left[140,280\right]$	Fitted
*Simulations*			
Time step	*dt*	1 day	
First day of trapping	$t_{0}$	1st June	
Last day of trapping	$t_{\mathrm{end}}$	31st January	

### 3.2 Scenario Runs

Using the 300 posterior parameter sets from the ABC model fitting, we ran 10,000 simulations for each of the seven scenarios (six lure combinations and one single‐lure baseline) described in the methods. The lure combination scenarios simulate the effects of introducing a secondary lure with an attractiveness potential independent to that of the baseline lure, and the advantages of using both lure types at the same time versus switching from one to the other once a given threshold is met. Each simulation was run using a randomly drawn parameter set from the 300 accepted sets. Results (Table 3) show a significant additional reduction in the surviving population after 8 months of trapping for all combination‐lure scenarios compared to the reduction achieved by the single‐lure baseline. Within the combination lure scenarios, those that had both lures for the entire duration of the programme resulted in the highest reduction in population size after 8 months, compared to the scenarios where the secondary lure was introduced only later to target the last trap‐shy individuals.

**TABLE 3:** Population size and mean $p_{\mathrm{int}}$ with lure A of the final population after 8 months of trapping, for both the baseline scenario and each combination lure scenario. Results show a median over 10,000 simulation repetitions and 95% credible intervals.

Scenario	Surviving population	Mean $p_{\mathrm{int}}$ towards lure A of surviving population ($\mu_{0}=0.39)$
Lure A (baseline)	50 [10, 152]	0.0008 [0.0001, 0.0029]
Lure A + B from $t_{0}$	21 [1, 106]	0.0012 [0.0000, 0.0074]
Lure A until $t^{*}$, then lure B (low change threshold—80% drop in daily captures)	29 [3, 118]	0.0230 [0.0005, 0.0888]
Lure A until $t^{*}$, then lure B (high change threshold—95% drop in daily captures)	26 [2, 114]	0.0134 [0.0005, 0.0575]
Lure A + C from $t_{0}$	7 [1, 50]	0.0009 [0.0000, 0.0210]
Lure A until $t^{*}$, then lure C (low change threshold—80% drop in daily captures)	8 [1, 52]	0.0165 [0.0000, 0.1336]
Lure A until $t^{*}$, then lure C (high change threshold—95% drop in daily captures)	7 [1, 55]	0.0101 [0.0000, 0.0817]

As expected, using a lure corresponding to a distribution of $p_{\mathrm{int}}$ with double the mean as that of the default lure produced to the strongest effect, with only 5% of the initial population left uncaptured after 8 months. Table S1 also shows a summary of the timing when the most trappable individuals have been caught and daily captures plateau, with the combination lure scenarios plateauing slightly later than the single lure one, but at much lower population numbers. All simulated scenarios resulted in a very low mean probability of interaction given encounter in the surviving population.

## 4 Discussion

Using lures in kill‐trap devices can increase wild animals' trappability, and a combination of lures would cater to diverse dietary preferences, potentially reducing the effort required for eradication. However, the extent and distribution of trap‐shyness in wild populations, and the optimal timing and choice of lures remain largely unknown.

We have used a spatially explicit model of population dynamics during a kill‐trapping operation, fitted to a brushtail possum capture dataset from a New Zealand forest reserve, to estimate the population's distribution of the trap‐shyness trait. Our model‐fitting results indicated that the possum population in the reserve was mostly comprised of a group of highly trap‐shy individuals and a slightly smaller group of highly trappable ones. The best‐fitting posterior distributions of the probability $p_{\mathrm{int}}$ of interaction with a device, given encounter, were U‐shaped (Figure 2d). This suggests a split in the population, with most individuals either consistently displaying trap‐shyness (very low $p_{\mathrm{int}}$) or getting trapped immediately (very high $p_{\mathrm{int}}$). Our estimates of the initial population size suggested that most of the population had been caught by the end of the 4‐month long trapping operation.

We then ran several scenarios considering different combinations of lures and different timings for the introduction of a second lure, applying the best‐case scenario assumption that animals' reactions to different lures are independent. All these scenarios resulted in a very low mean probability of interaction given encounter $p_{\mathrm{int}}$ in the surviving population, which highlights the vexing problem that pest managers face—that the surviving animals left at the end of a trapping operation are typically the most trap‐shy individuals. Our results also suggested that having a mix of both lures for the entire duration of the trapping operation is more effective than switching from one lure to another part way through the operation. Regardless of the lure combination used, there will still be some very trap‐shy individuals that remain uncaught. These last trap‐shy survivors could be targeted in a number of ways, depending on our assumptions on their behaviour. If we assume that individual attraction towards a specific lure is completely independent from their attraction towards other lures, the best strategy would be to keep introducing additional lures to try and cater to every individual taste. However, if animals' trap‐shyness is somewhat independent of the lure used, introducing additional lures will have no effect on the surviving population, which will instead need to be eradicated using more intensive methods. Real‐world populations probably display a combination of these factors: introducing a new lure will capture a portion of the remaining survivors, but the most trap‐shy individuals will remain uncaptured. The ideal management strategy would achieve the fastest knockdown in the shortest timeframe, which minimises the opportunity for pests to breed and can trigger the application of alternative strategies to target surviving trap‐shy individuals. For example, one could use lured traps to reduce the population to the last few trap‐shy individuals, and then use other devices (such as camouflaged leg‐hold traps or dogs), whose efficacy is independent of the possums' kill‐trap response. These findings are relevant to any control scenario where invasive mammalian pests are lured into devices.

Our modelling study, and the underpinning field data, illustrate why trapping is unlikely to achieve predator eradication unless supplemented with other control methods. First, as shown by the model calibration stage of our study, average $p_{\mathrm{int}}$ for a widespread predator population will typically be well below 0.5, which means that even in the absence of capture heterogeneity it will take weeks or months to substantially reduce the population. This creates a ‘long tail’ effect whereby the per capita effort required to capture or kill further predators rises exponentially (see figure 1 in Johnstone, Garvey, and Hickling 2023). Second, capture heterogeneity exhibited by real‐word predator populations makes it particularly hard to capture individuals from post‐control residual populations, and improved luring methods offer a potential way to address this problem. In a ‘best case’ scenario (as modelled here, with different lures operating independently in attracting different subpopulations of predators) the use of a second lure can substantially reduce—although still not eliminate—the surviving population (see Table 3). The advantage of using multiple lures will be reduced, however, if the multiple luring effects do not operate independently; that is, if the animal is strongly attracted to one lure they also tend to be attracted to the other lure. Currently, there are almost no available data on the dependence/independence of predator' responses to multiple lures, but reliance on a single control method to suppress or eradicate an entire population is unlikely to be an effective strategy to target the most trap‐shy individuals, resulting in slow progress or even operational failure (Amos et al. 2016; Cowan 1992). We suggest, however, that the best prospects for achieving an additive effect of multiple lures may come from combining lures that target different sensory modalities. For example, a lure that combines auditory, visual and olfactory stimuli may succeed in attracting a greater number of trap‐shy individuals than a lure that combines, say, three different olfactory cues. Testing this hypothesis could be a helpful next step in developing more effective multilure strategies for invasive predators. Finally, pests can exhibit resistance to control by toxic baits due to innate and learned aversion (Allsop et al. 2017). Bait additives can mask signals used by target animals and disrupt the development of learned bait aversion. In line with our findings, switching between bait types has been recommended to reduce target species becoming habituated to avoiding baits (Allsop et al. 2017), and providing a range of options can reduce the impact of learned aversion, while also reducing the impacts of innate individual preferences.

The model described in this paper has some important limitations that need to be considered. Because of the short time frame modelled (8 months starting on 1st June), we assumed a closed population, with the only driver of population size being density‐dependent reproduction. We do not model immigration from external populations or range expansion by neighbouring possums, which are known to be an important factor in longer‐term eradication operations. In the scenarios with both lures present simultaneously, we do not explicitly model which traps are lured with either lure type; instead, we assume an equal probability of encountering a trap with either lure. This is a reasonable assumption for scenarios with only two lure types and with a high density of traps, however our results might not apply in scenarios with low trap densities or with a higher number of lure types, where lures would have to be regularly swapped between traps to allow encounters by each individual. In addition, our assumptions on the attractiveness of the second lures were purely arbitrary, as little is known about brushtail possums' relative levels of attraction towards different lures. Independence of shyness levels across different devices has been observed in rats (Johnstone, McArthur, and Banks 2021), and the variable effects of scent lure as attractants across species are well understood (e.g., Holinda, Burgar, and Burton 2020), yet there have been no studies on heterogeneity of attraction based on individual behavioural traits. Future work should focus on conducting field or pen trials where individual behaviour towards kill traps is repeatedly tested using different lure compounds, to justify our assumptions of a consistent individual behavioural response to a particular lure, and of independence of attraction levels towards different lures. Our model also assumed no change in trap‐shyness after a failed interaction with a trap (e.g., a trap is triggered but the animal escapes). These events are relatively common for possums (Warburton 1982) and other invasive predators (Weihong, Veitch, and Craig 1999), and can significantly increase individual trap‐shyness, leading to a population that is more difficult to eradicate. In such a scenario, alternative lures are unlikely to override the learned shyness, and changes in trap type or to passive control methods will be required.

## Author Contributions

**Giorgia Vattiato:** conceptualization (equal), formal analysis (equal), methodology (equal), software (equal), visualization (equal), writing – original draft (equal), writing – review and editing (equal). **Patrick M. Garvey:** conceptualization (equal), data curation (equal), methodology (equal), project administration (equal), supervision (equal), writing – original draft (equal), writing – review and editing (equal). **Rachelle N. Binny:** conceptualization (equal), methodology (equal), supervision (equal), writing – review and editing (equal). **Michael J. Plank:** conceptualization (equal), methodology (equal), supervision (equal), writing – review and editing (equal). **Andrew M. Gormley:** conceptualization (equal), writing – review and editing (equal). **Graham J. Hickling:** conceptualization (equal), data curation (equal), methodology (equal), project administration (equal), supervision (equal), writing – original draft (equal), writing – review and editing (equal).

## Conflicts of Interest

The authors declare no conflicts of interest.

## Declaration

Our study was based on theoretical simulation, and analysis of secondary data rather than primary data. As such, there was no local data collection. All authors are based in the country where the study was carried out and were engaged early on with the study design to ensure that the diverse sets of perspectives they represent was considered from the onset. Whenever relevant, literature published by scientists from the region was cited.

## Supporting information


Data S1

## Data Availability

The dataset used to calibrate our model is publicly available through the Manaaki Whenua Landcare Research datastore repository (https://doi.org/10.7931/6y1w‐s375). The simulation model code is publicly available on Zenodo (https://doi.org/10.5281/zenodo.14015234).
