# Supplementary material for: Optimising Control Device Luring Strategies for Invasive Predator Control: A Modelling Approach
Source: Ecol Evol. 2024 Nov 28;14(12):e70604. doi: 10.1002/ece3.70604 (PMC11603212; doi:10.1002/ece3.70604)
Supplement: Supplementary file 1 — Data S1 [file ECE3-14-e70604-s001.docx]

# SUPPLEMENTARY MATERIAL

**Daily captures plateau and timing to change surveillance**

When trying to eradicate a behaviourally heterogeneous population with trap-shy individuals, the number of daily captures diminishes over time and eventually plateaus as the more trappable individuals get caught and the surviving population becomes less and less trappable. Supp. Table S1 shows an analysis of the time $t^{*}$ when daily captures drop by a given threshold and start plateauing, as well as the proportion of the original population still surviving at time $t^{*}$ and its mean interaction probability towards standard lure A.

Using the U-shaped posterior distributions of probability of interaction with the traps, presented in the main Results section of this paper, our simulations showed that regardless of the lure combination used, daily captures drop by 80% within 14 days of the start of the operation, and by 95% within 30 days, with the two-lure combination scenarios showing a slightly longer time before the daily capture plateau. However, using a combination of lures from the start resulted in a bigger proportion of population getting trapped within that first period.

This type of analysis can inform future cost-benefit studies exploring the best timing to change surveillance strategy from trapping to a more effective – and potentially more costly – one, such as the use of possum-detecting dogs or thermal-camera drones.

Supplementary Table S1 – Time $\boldsymbol{t}^{\boldsymbol{*}}$, proportion of original population still surviving, and mean probability of interaction with lure A $\boldsymbol{p}_{\boldsymbol{int,A}}$ of the survivors when daily captures drop by a set amount (low threshold – 80%, high threshold – 95%), for different combination of lures used at the same time from $\boldsymbol{t}_{\boldsymbol{0}}\boldsymbol{=0}$. See the Methods section of the main text for details on the three different lures. Time $\boldsymbol{t}^{\boldsymbol{*}}$ corresponds to the time when daily captures start levelling off due to the low capture probability of the surviving population. The drop in daily captures is calculated by taking the ratio of the current daily catch rate (averaged over the past seven days, with the earliest possible threshold time being $\boldsymbol{t}^{\boldsymbol{*}}\boldsymbol{=6}$ days) over the number of captures at $\boldsymbol{t=1}$ (after the first day of trapping). Results show the median over 10,000 simulation repetitions and the 95% credible intervals.

| Scenario | Time $\boldsymbol{t}^{\boldsymbol{*}}$ when threshold is met (days since beginning of trapping) | Proportion of original population surviving at $\boldsymbol{t}^{\boldsymbol{*}}$ | Mean $\boldsymbol{p}_{\boldsymbol{int,A}}$ of surviving population at $\boldsymbol{t}^{\boldsymbol{*}}$ |
| --- | --- | --- | --- |
| *Lure A* |  |  |  |
| *Low threshold*  *(80% drop in daily captures)* | 6 [6, 11] | 57% [29%, 76%] | 0.026 [0.003, 0.068] |
| *High threshold*  *(95% drop in daily captures)* | 10 [7, 26] | 52% [24%, 75%] | 0.016 [0.002, 0.045] |
| *Lure A+B* |  |  |  |
| *Low threshold*  *(80% drop in daily captures)* | 8 [6, 14] | 39% [13%, 65%] | 0.051 [0.013, 0.154] |
| *High threshold*  *(95% drop in daily captures)* | 14 [9, 30] | 32% [9%, 60%] | 0.025 [0.005, 0.098] |
| *Lure A+C* |  |  |  |
| *Low threshold*  *(80% drop in daily captures)* | 7 [6, 12] | 22% [10%, 45%] | 0.051 [0.007, 0.167] |
| *High threshold*  *(95% drop in daily captures)* | 12 [9, 25] | 16% [6%, 38%] | 0.028 [0.002, 0.121] |


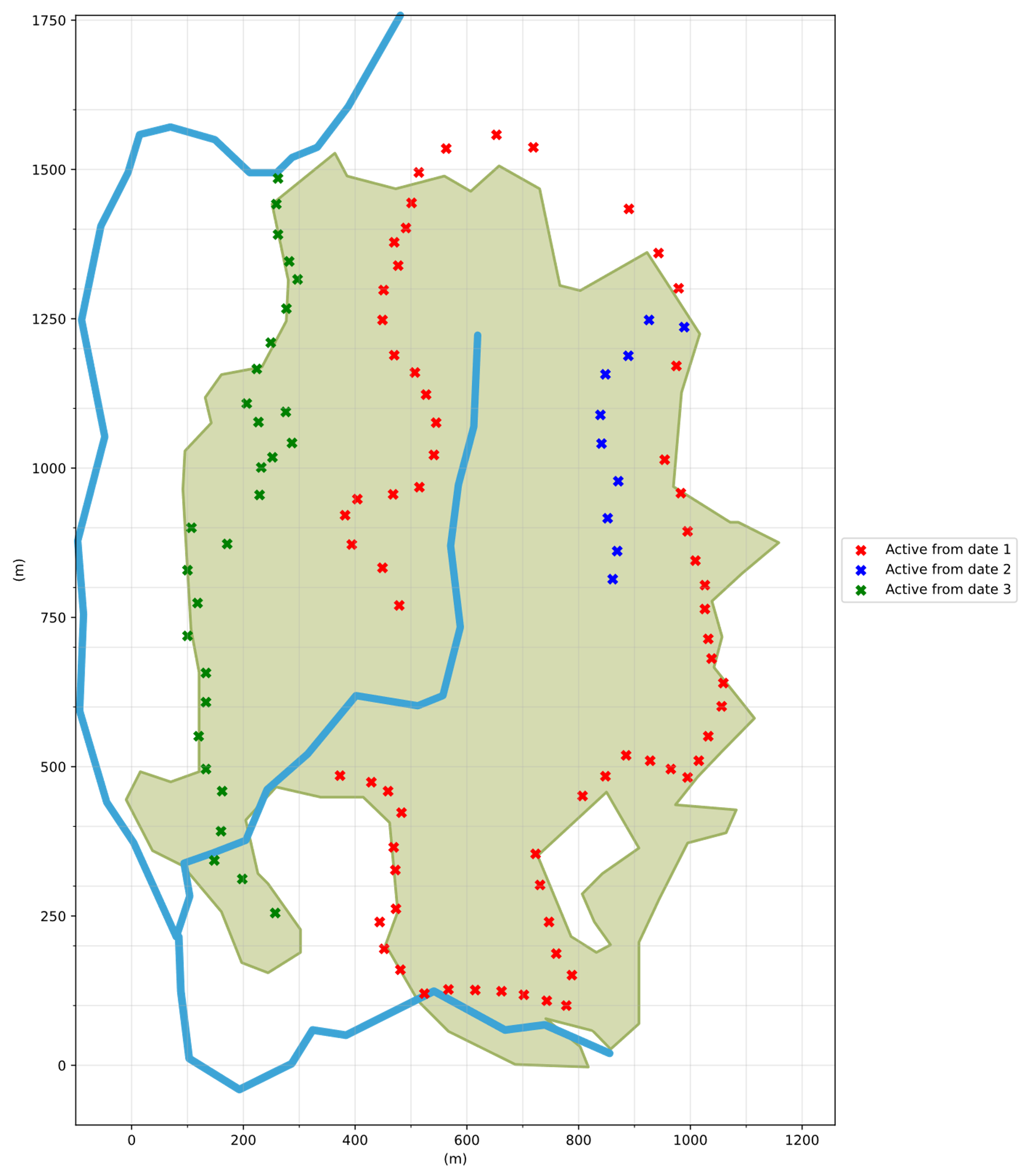


Supplementary Figure S1 – Trap layout at the Lottery Bush capture dataset (Johnstone et al., 2023); this layout was replicated in the simulation model we fitted to that dataset. Each square corresponds to one hectare, and each coloured cross corresponds to one of the three sets of traps activated at different dates: in red, the 66 traps activated on the 3^rd^ May (beginning of simulation); in blue, the additional 10 traps activated on the 9^th^ June; in green, the additional 29 traps activated on the 15^th^ June. Green shading indicates the extent of the relatively isolated patch of forested possum habitat (Johnstone et al., 2023), the blue lines correspond to rivers, which helped to minimise incursions of possums from outside the reserve..


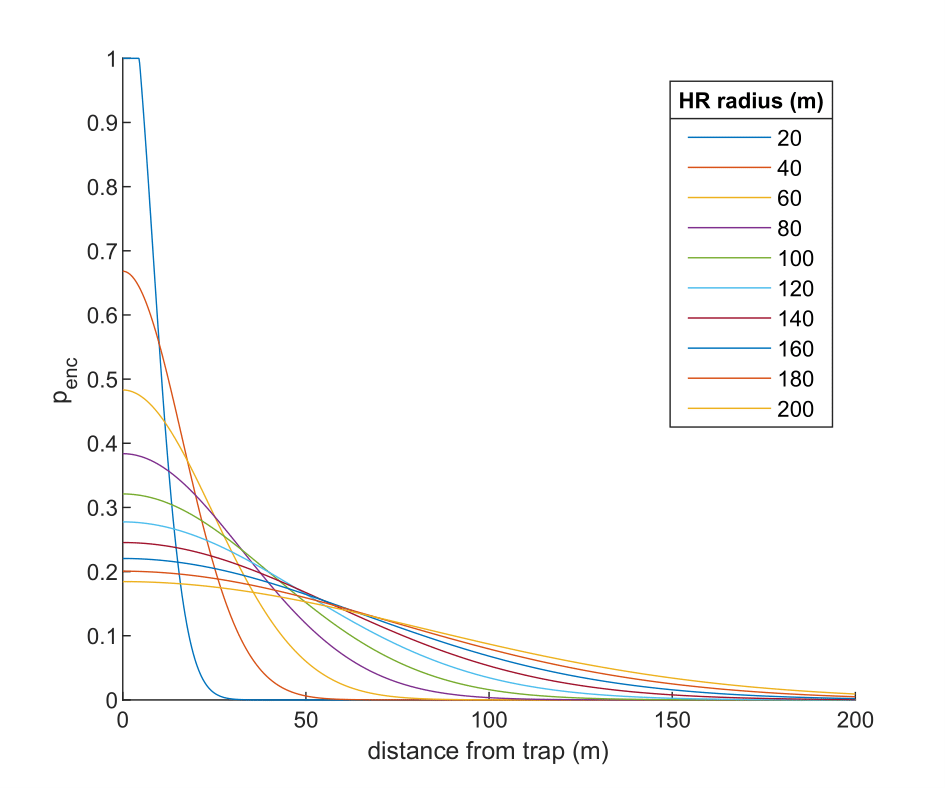


Supplementary Figure S2 - Relationship between the probability of encounter $\boldsymbol{p}_{\boldsymbol{enc}}$ and the distance $\boldsymbol{d}$ between trap and home-range centre. Plots produced using Equation 6 for different values of $\boldsymbol{\sigma}$ (which was converted to HR radius using the formula $\boldsymbol{\sigma=}\mathbf{HR radius/}\boldsymbol{2.45}$, in accordance with the definition of $\boldsymbol{\sigma}$).


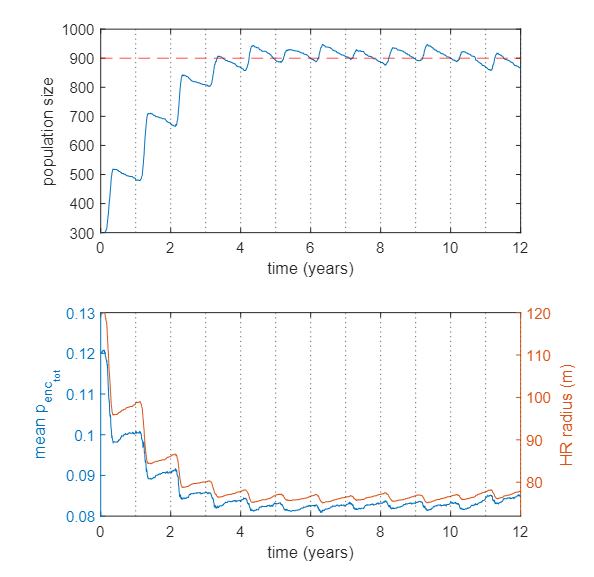


Supplementary Figure S3 - Population dynamics with seasonal reproduction and natural mortality in the absence of trapping, simulated using parameter values in Table 1 and for an initial population $\boldsymbol{N}_{\boldsymbol{0}}\boldsymbol{=300}$. Top: Change in population size over time. The red horizontal line corresponds to carrying capacity. Bottom: Change in mean total probability of encounter $\boldsymbol{p}_{\boldsymbol{encTOT}}$ (blue; as defined in Equation 7) and density-dependent home-range radius (red) over time. The reduction in encounter probability is a direct consequence of the reduction in home-range radius.


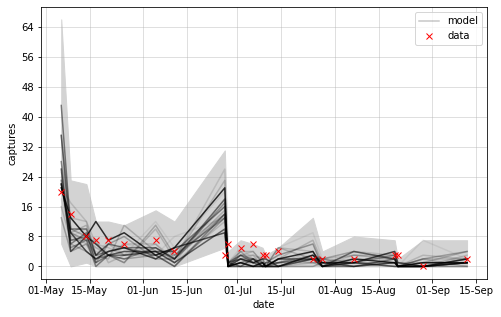
Supplementary Figure S4 – Comparison of capture data (red crosses) from the Lottery Bush experiment (Johnstone et al., 2023), and the modelled captures for those same time periods. Graph shows the curvewise 95% credible interval (grey shaded area), and a sample of 20 model trajectories (solid grey line), with a transparency gradient which reflect the goodness of fit of each trajectory (the darker the line, the better the fit to the data).

**References**

Johnstone, K., Garvey, P., & Hickling, G. (2023). Invasive mammal control selects for trap-recalcitrant behaviour and personality. *Biological Invasions* 1-16. <https://doi.org/https://doi.org/10.1007/s10530-023-03191-4>
